# Coherent interaction of atoms with a beam of light confined in a light cage

**DOI:** 10.1038/s41377-021-00556-z

**Published:** 2021-05-31

**Authors:** Flavie Davidson-Marquis, Julian Gargiulo, Esteban Gómez-López, Bumjoon Jang, Tim Kroh, Chris Müller, Mario Ziegler, Stefan A. Maier, Harald Kübler, Markus A. Schmidt, Oliver Benson

**Affiliations:** 1 Department of Physics and IRIS Adlershof, Humboldt-Universität zu Berlin, 12489 Berlin, Germany; 2 Chair in Hybrid Nanosystems, Nanoinstitute Munich, Faculty of Physics, Ludwig-Maximilians-Universität München, 80539 Munich, Germany; 3 Department of Fiber Photonics, Leibniz Institute of Photonic Technology, 07745 Jena, Germany; 4 Competence Center for Micro- and Nanotechnologies, Leibniz Institute of Photonic Technology Jena, 07745 Jena, Germany; 5 Department of Physics, Imperial College London, London, SW7 2AZ UK; 6 5. Physikalisches Institut and Center for Integrated Quantum Science and Technology, University of Stuttgart, Pfaffenwaldring 57, 70569 Stuttgart, Germany; 7 Otto Schott Institute of Material Research, 07743 Jena, Germany

**Keywords:** Integrated optics, Atom optics, Polymers

## Abstract

Controlling coherent interaction between optical fields and quantum systems in scalable, integrated platforms is essential for quantum technologies. Miniaturised, warm alkali-vapour cells integrated with on-chip photonic devices represent an attractive system, in particular for delay or storage of a single-photon quantum state. Hollow-core fibres or planar waveguides are widely used to confine light over long distances enhancing light-matter interaction in atomic-vapour cells. However, they suffer from inefficient filling times, enhanced dephasing for atoms near the surfaces, and limited light-matter overlap. We report here on the observation of modified electromagnetically induced transparency for a non-diffractive beam of light in an on-chip, laterally-accessible hollow-core light cage. Atomic layer deposition of an alumina nanofilm onto the light-cage structure was utilised to precisely tune the high-transmission spectral region of the light-cage mode to the operation wavelength of the atomic transition, while additionally protecting the polymer against the corrosive alkali vapour. The experiments show strong, coherent light-matter coupling over lengths substantially exceeding the Rayleigh range. Additionally, the stable non-degrading performance and extreme versatility of the light cage provide an excellent basis for a manifold of quantum-storage and quantum-nonlinear applications, highlighting it as a compelling candidate for all-on-chip, integrable, low-cost, vapour-based photon delay.

## Abstract (web summary)

Light-matter interaction in the hollow-core light cage, at the example of EIT in alkali atoms in this work, presents a novel approach to implement integrated on-chip devices for applications in quantum optics and quantum information.

## Graphical abstract

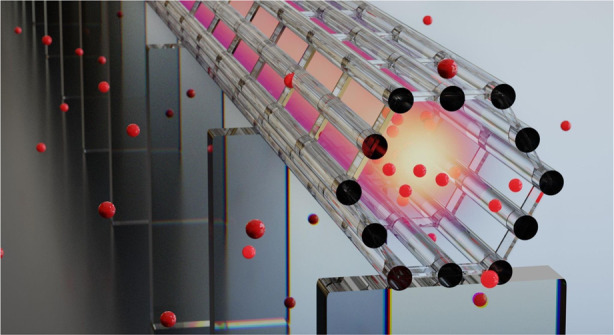

## Introduction

In the rapidly growing field of hybrid quantum photonics, the realisation of miniaturised, integrated quantum-optical systems with small geometric footprints and intense light-matter interaction is of great importance for both fundamental and applied research^1–8^. Specifically, the development of methods to reliably generate, control, store and retrieve quantum states with high fidelity through coherent interaction of light and matter, unveiled a vast field of applications for quantum information and quantum networks such as optical switching^9^, photon routing with isolators^10^ or circulators^11^, entanglement generation^12^ and distillation^13^, quantum memories using cold^14^ or warm^15^ atoms, and quantum repeaters, in order to achieve truly scalable networks^16^.

One promising approach for efficient light-matter interaction relies on integration of light-guiding platforms in near-room-temperature alkali vapour, as presented in this work. Several research groups have aimed to integrate hollow-core photonic-crystal fibres (HC-PCFs)^17–22^ or planar waveguides^23–27^ with atoms in vapour cells. When coupled to atoms, nearly all photonic structures, however, reveal distinct limitations imposed by their design: (i) HC-PCFs and on-chip hollow-core antiresonant reflecting optical waveguides (ARROWs)^28^ are capillary-type structures that require vapour-filling times exceeding months for few centimetre long devices^20,29^. Waveguides with exposed evanescent fields partially overcome this issue, while the limited spatial extend of the field (<1 µm) induces (ii) large decoherence, and (iii) low atomic-vapour densities near the surfaces of the waveguide^30^. It is possible to increase the optical density by using either tapered fibres in combination with cold atoms in cryogenic environments^31^ or light induced atomic desorption (LIAD)^29,32,33^. Nonetheless, these approaches induce an unwanted level of complexity, especially when aiming for integrated applications. The infinite, diffraction-free ideal Bessel beam^34^ could also be regarded as a candidate for extended light-matter interaction. In practice, however, the ideal case can only theoretically be reached and losses of intensity along the propagation axis are to be expected^35^. It is also noteworthy that using a glass axicon to produce the Bessel beam would induce chromatic aberration which in turn, would require special adaptation in experiments at distinct wavelengths^36^. Another concern when using Bessel beams is their prominent lobes where a considerable proportion of the overall optical power is distributed across the outer rings, creating substantially different intensities for light-matter interaction compared to the central lobe. Moreover, to reach beam lengths of around 20 mm^37^, bulky optical systems with transverse extension in the millimetre range would be required, rendering fibre integration impracticable.

**Fig. 1:**
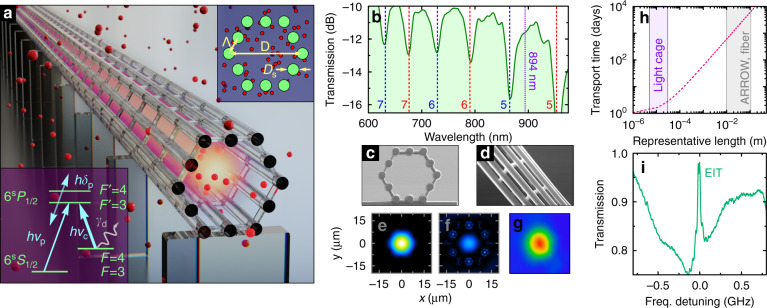
Quantum-optically integrated light cage. **a** Sketch of the light cage (LC) enclosing a light beam (yellow) exposed to caesium (Cs) vapour (red spheres). Bottom inset: EIT Λ-scheme with probe (*ν*_p_) & coupling fields (*ν*_c_) and coherence dephasing *γ*_d_ of the F = 4 hyperfine state. Top inset: cross section of the LC geometry including design parameters (*D*_s_ = 3.6 μm, *Λ* = 7 μm, *D* = 28 µm, and 4.5 mm length). **b** Transmission spectrum of the Cs-cell-integrated LC in the visible–near-infrared with the Cs-D1 line (894 nm) in a leaky mode between cut-off frequencies of isolated strand modes^38,39^ (dashed lines, blue: LP_0x_, red: LP_1x_). **c** Scanning-electron microscopic image of the LC front facet. **d** Side view of the sample, showing open spaces between strands. Simulated spatial field distributions, **e** within a transmission band (894 nm) and, **f** near a resonance (930 nm). **g** Measured output field pattern. **h** Improvement of filling time, revealed by plotting the transport time versus representative length. Three orders of magnitude faster filling is provided by the LC compared to capillary-type waveguides. (Details in Supplementary Section II.) **i** Example of a measured EIT spectrum

To circumvent the drawbacks of conventional hollow-core waveguidance while maintaining a compact, easy-to-handle light-guiding structure, the recently introduced on-chip hollow-core waveguide—the light cage (LC)^38–40^—was adopted as vessel for coherent light-matter interaction. Here, we exploit in particular the properties of the non-diffractive nature of the anti-resonant leaky mode inside the hollow core^41^ with side-wise access over millimetre distances, which essentially result in a cylindrical beam with an intensity distribution resembling a *rod of light*. Note that the non-zero power loss from the leaky LC mode prevents guidance over lengths exceeding tens of centimetres while, however, propagation across several centimetres^42^, as recently demonstrated, is compatible with applications such as the one presented in this manuscript. The polymer structure consists of a ring of high-aspect-ratio, micrometre-size strands circumscribing a side-wise accessible hollow core (Fig. 1a, c, d). Implemented by 3D nanoprinting^43,44^, the LC supports core modes via the anti-resonant effect and exhibits unique features: (I) side-wise direct access to the hollow core through the interstices between the strands for high-speed gas diffusion (Supplementary Section II and Fig. 1h), (II) exceptionally high fraction of modal fields in the hollow section (99.9%, Fig. 1e), and (III) diffraction-less propagation over millimetre distances. Moreover, the resistance of LCs to corrosive substances was substantially improved via nanofilm coating^39^, (IV) making the LC concept particularly attractive for alkali vapour-based experiments.

In this article, we demonstrate the peculiar coherent quantum-optical interaction between the light field in a LC and room-temperature caesium (Cs) atoms by the observation of modified electromagnetically induced transparency (EIT, example spectrum in Fig. 1i). Compared to other hollow-core waveguide structures, the LC uniquely provides low decoherence^20^ due to minimal atom-wall collisions. Moreover, the high degree of integration and long-term stability allows for interfacing our device with other established technology platforms like silicon photonics and fibre optics, as well as customising reproducible devices for a large variety of applications. The measurements and simulations presented here reveal a non-intuitive yet profitable quantum-optical behaviour stemming from strong light confinement over long distances, thus confirming the enhancement of light-matter interaction within the LC.

## Results

### Rod of light in a vapour-filled light cage

The LC was fabricated by 3D nanoprinting (Nanoscribe GT) of a polymer (IP-Dip) on a silicon substrate (details in Methods and Supplementary Section I). To protect the printed LC structure from Cs-induced chemical degradation, the structures were coated with a 100 nm thick alumina layer via low-temperature plasma-enhanced atomic layer deposition (PE-ALD). Note that the layer deposition allows for fine-tuning of the spectral properties (i.e. position of low transmission resonances) with high precision^39^. The layer thickness used here is chosen such that the Cs D1 absorption line at *λ*_Cs D1_ = 894 nm coincides with a high-transmission window (Fig. 1b), while also providing sufficient chemical protection. The printed chip was placed inside a custom-made hermetically sealed glass cell and filled with Cs atoms by thermal diffusion (details in ‘Methods’ section). The beam is confined in the light cage with a mode diameter of 12.6 µm over a length of 4.5 mm. Light confinement and guidance inside the cell-integrated structure were confirmed by the spectral distribution of the transmission (Fig. 1b, vapour-exposed structure, details in the Supplementary Section I). Indeed, the presence of anti-resonance guidance is clearly shown with the observation of a series of high transmission windows separated by resonances and by measuring the mode profile at the output of the LC as seen in Fig. 1g. The modal attenuation was found to be in the order of *α*_LC_ ≈ 1 dB mm^−1^ at the target wavelength of 894 nm (Supplementary Section III). Long-term chemical and mechanical stability were confirmed by comparing the designed cut-off wavelengths of strands (dashed red and blue lines in Fig. 1b) with the measured resonance positions (green curve), 10 months after the vapour-cell integration, showing no visible change of the optical properties. Furthermore, an atom-number density of the order of 10^11^ cm^−3^ was reached, close to the result in ref. ^29^. Remarkably, such densities are obtained without additional desorption processes^29,33^ or paraffin cell coating^32^.

### Observation of EIT in the light cage

**Fig. 2:**
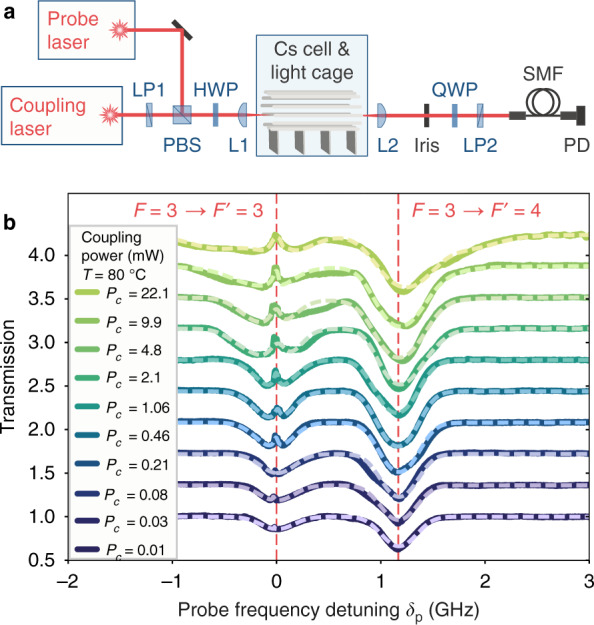
EIT in the light cage (LC). **a** Setup for the optical experiments. The probe and coupling laser beams are jointly coupled into the LC (placed inside the temperature-controlled Cs vapour cell) with an aspheric lens (L1) and recollimated afterwards (L2). Polarisation optics are used to suppress the coupling laser (linear polarisers LP1, LP2; polarising beam splitter PBS; half- and quarter-wave plate HWP, QWP). An iris and single-mode fibre (SMF) coupling ensure spatial filtering before detection on a photo diode (PD). **b** Broadening and additional absorbing shoulders near the EIT peak appear at high coupling laser powers. Dashed lines are fitting curves

Electromagnetically induced transparency (EIT) results from an interference effect in a three-level system^45^. The typical scenario is a Λ-system (bottom inset in Fig. 1a) where absorption and dispersion of a probe-laser beam are substantially modified by the presence of an additional strong pump- or coupling-laser beam. A key feature is the appearance of a narrow transparency peak in a broad absorption dip with increasing power of the coupling laser (see Fig. 1i). In our experiment we use the optical D1 transitions of Cs atoms near 894 nm. EIT is established for the probe laser on resonance (*δ*_p_ = 0 GHz, lower inset in Fig. 1a) in the Λ-system between the two hyperfine-split electronic ground states (F = 3 and F = 4) and the lowest excited state (F′ = 3) in the presence of a strong coupling laser, locked to the hyperfine transition F = 4 → F′ = 3 (*ν*_c_ in Fig. 1a, bottom inset). Probe and coupling lasers are overlapped and coupled into the LC located inside a Cs vapour cell with controllable oven temperature of *T* = 40 – 80 °C (Fig. 2a and ‘Methods’: Light-cage integration). A temperature just above room temperature is desired for straight-forward temperature stabilisation of the vapour cell with a simple oven that matches with the required temperature range for caesium vapour to reach a reasonable atomic density in a millimetre-long cell. Inside the LC both laser fields propagate diffraction-less and can strongly interact with the in-diffusing atoms. The coupling laser is up to four orders of magnitude more intense than the probe laser at *P*_p_ = 5 μW. The probe and coupling fields are only detuned by *ν*_p_ − *ν*_c_ = 9.193 GHz in case of on-resonant EIT. Masking of the probe laser upon detection is avoided by polarisation filtering of the strong coupling field.

Figure 2b shows measurements of the probe transmission for different intensities of the coupling laser at an oven temperature of *T* = 80 °C. The intensity of the coupling laser in the LC was changed over three orders of magnitude; from *I*_c_ = 8 W cm^−2^ to 24 kW cm^−2^. With increasing power of the coupling laser an EIT transmission peak appears at resonance. The very large range of coupling-laser powers allowed for examination of three regimes of EIT in the LC. For coupling powers near the probe power, *P*_p_ = 5 μW, we observe a strong probe regime: it manifests as a more triangular lineshape of the F = 3 → F′ = 4 resonance at *δ*_p_ = 1.17 GHz and can be explained as reduced effective coupling Rabi frequency^46^. In the intermediate range, at *P*_c_ ≈ 0.2 mW, the coupling field is much stronger than the probe field, but still weak enough to produce a spectrum closely resembling that of ideal EIT, i.e. a transmission peak within an otherwise unaltered absorption spectrum. In the strong coupling-laser regime, an augmentation in the transmission-peak width is witnessed as the coupling power is increased^47,48^. However, the peculiarity of this regime is the experimental observation of absorbing shoulders at *δ*_p_ ≈ −0.3 GHz and 1.5 GHz.

### Comparison of EIT in the light cage and in free space

**Fig. 3:**
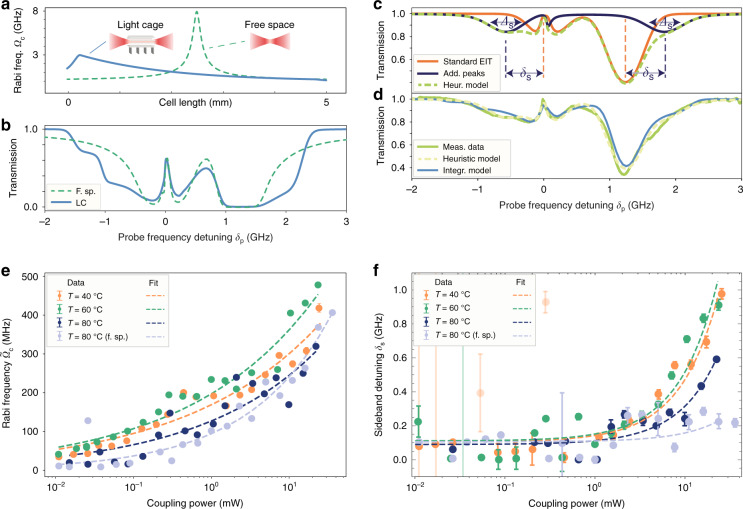
Comparison of EIT in the LC and free space. **a** Coupling-field amplitude along the propagation axis, expressed as Rabi frequency Ω_c_(*z*), for the free-space (dashed line) and LC (solid line) situation. **b** Calculated transmission spectra for free space (dashed) and the LC (solid). **c** Heuristic fitting curve (dashed, example: *P*_c_ = 22.1 mW, *T* = 80 °C) decomposed into an analytic EIT curve (orange) and sidebands (blue) with relative detuning *δ*_S_ and width *∆*_S_. **d** Comparison of heuristic fitting, numerical effective curve and measured data. **e** Effective Rabi frequencies $\tilde \Omega _c$ in the LC and free space (f.sp.) derived from the data as a function of coupling-laser power *P*_c_ for different oven temperatures (error bars smaller than ticks). The dashed lines are fits ∝(*P*_*c*_)^*m*^ with an average exponent $\bar m_{{\mathrm{LC}}} = (0.26 \pm 0.05)$ in the LC and *m*_fs_ = (0.41 ± 0.04) in free space (errors are the standard deviation of the fit), respectively. **f** Obtained detunings *δ*_s_ of the absorbing sidebands in the LC and free space (f.sp.) as a function of coupling laser power for different oven temperatures

In the following, we discuss the measured EIT spectra in the LC and compare them with a free-space analogous situation. There is a notable difference between these two cases. In free space, both beams are tightly focused Gaussian beams. Along the propagation axis, the field amplitude is rapidly increasing before the focal point and rapidly decreasing thereafter. In the LC, the light is focused at the input of the structure, then propagates as confined mode without diffraction and finally diverges from the output facet. Different losses apply to the two experiments compared here (see Supplementary Section III). However, the deviation of the fibre-coupling efficiencies from ideal 100 % and the normalisation of the transmission spectra (see Supplementary Section IV) do not affect the measured spectral features. This is due to the fact that the transmitted probe power only undergoes constant loss over the scanning range after passing the vapour cell. Figure 3a shows the Rabi frequency Ω_c_ corresponding to the coupling-beam amplitude for the free-space (dashed line) and LC (solid line) situation, respectively. Please note that the exponential decrease for the LC curve corresponds to scattering losses of the LC. Our theoretical model of EIT accounts for an explicit *z*-dependency since integration along the propagation axis is performed (refer to Supplementary Section IV). To emphasise the absorptive shoulders more in the calculated transmission spectrum, it is plotted in Fig. 3b as solid blue line with a high effective vapour temperature *T*_vap_ = 80 °C that includes the inhomogeneous broadening of the absorption and a higher atom density, which is also used as basis for the delay simulation in the next section. In the experiment a reduced vapour density is observed compared to the expected densities at the temperatures measured close to the heaters. This can be explained as an effect of deposition of atoms on the LC and the vapour cell walls, together with thermal dissipation from the heaters to the sample area resulting in a lower temperature of the LC. However, no additional efforts were necessary to reach sufficient vapour densities for the present experiments. The theoretical curve, taking into account this reduced vapour density, accurately reproduces the experimental data, as shown in Fig. 3d. In particular, it confirms the presence of additional absorbing shoulders near the EIT peak, which cannot be explained by thermal or power broadening alone. More modified spectra are plotted in Supplementary Fig. S2 for varying initial Rabi frequencies. In order to simplify the fitting of all data to theoretical curves, we introduce a heuristic transmission curve (see Supplementary Section IV for details), which is composed of two parts. One part is the ideal, temperature-broadened EIT spectrum as described above according to ref. ^45^. The other part consists mainly of two Gaussian sidebands of FWHM ∆_*s*_ and symmetric sideband detuning *δ*_s_ from the two atomic transitions (F = 3 → F′ = 3 and F = 3 → F′ = 4). We plotted this fitting curve and its two components in Fig. 3c, d as well (dashed green lines).

From the heuristic fitting curve, the effective coupling Rabi frequency $\tilde \Omega _c$ and the sideband detunings *δ*_s_ can be derived (Fig. 3e, f). These parameters reflect the strength of atom-field interaction in the LC as compared to free space. Data for additional temperatures in the LC and in free space are displayed in Supplementary Fig. S3. The comparison of the onset of the EIT dips at low powers and the nearly identical EIT-window widths between light cage and free space lead to the conclusion that the intensities present in the light cage are nearly identical to the free-space situation. This underlines the efficient coupling of the probe field into the light cage. The power dependency of the Rabi frequency (Fig. 3e) can be fitted with a free exponent *m* according to $\tilde \Omega _c \propto (P_c)^m$. For the free-space data this yields an exponent *m*_fs_ = (0.41 ± 0.04) close to the expected^45^ $\tilde \Omega _c \propto (P_c)^{1/2}$ behaviour, since the Rabi frequency should be proportional to the field amplitude. In the LC, the average exponent $\bar m_{{\mathrm{LC}}} = (0.26 \pm 0.05)$ over all three temperatures, is smaller than in the free-space situation. This is due to the attenuation in the LC used in this experiment and therefore the decreasing Rabi frequency along the propagation axis. In a LC with smaller losses, which can be achieved by an improved design^40^, the exponent would also approach *m*_LC_ = 1/2. At coupling powers below *P*_c_ = 10 mW, the light cage clearly outperforms the free-space situation. The sideband detunings *δ*_s_ (Fig. 3f) shift linearly with increasing coupling laser power (dashed lines, note the semi-logarithmic scale!). Outlying data points in the low coupling-power region were omitted from fitting, where the amplitude of the additional absorbing features was nearly zero.

The experimental results of EIT in the LC with its non-diffractive optical fields already indicate qualitative deviations from the free-space scenario. This is caused by a high intensity maintained along the whole propagation axis. With less scattering loss in the LC the intensity could even be kept constant over long distances, which leads to an improved performance as discussed in the next section.

### Decoherence properties of EIT in the light cage and prospects as quantum delay

The enhancement of light-atom coupling via confined light in waveguides is deteriorated by the interaction with nearby surfaces. Indeed, we also observed a slightly lower absorption of Cs resonances in the LC, indicating a reduced vapour density in comparison to free space (Supplementary Section II). However, we still achieved atomic densities on the order of 10^11^ cm^−3^. This is extraordinarily high as compared to capillary-type waveguides, where such densities can only be achieved by applying additional techniques, such as light induced atomic desorption (LIAD).

**Fig. 4:**
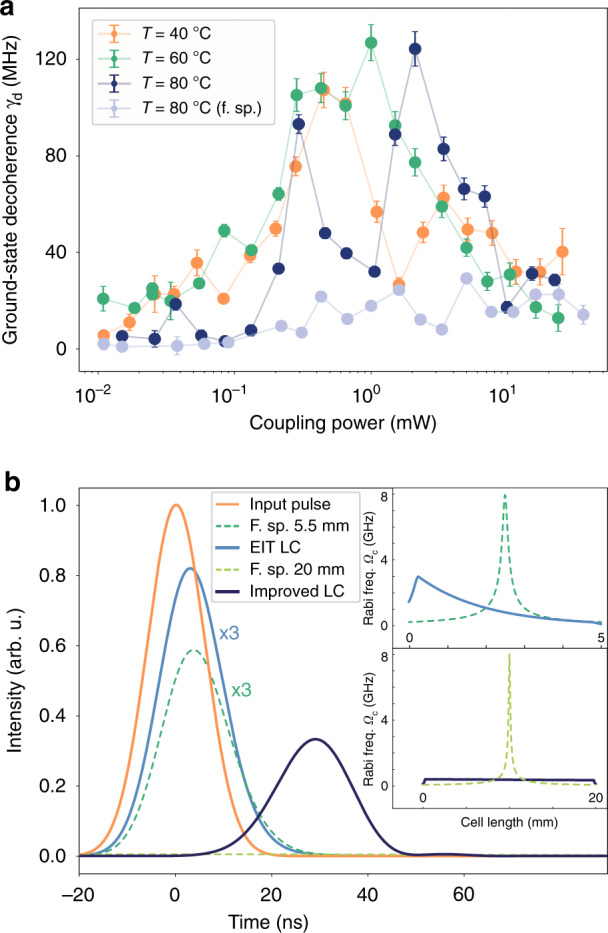
Decoherence in LC and prospects for EIT delay. **a** Derived ground-state decoherence *γ*_d_ as a function of the coupling laser power for different temperatures in the LC compared to free space (f.sp.). **b** Simulated transmission of a Gaussian light pulse with 14 ns width (orange) through the 4.5 mm long LC (5 mm vapour cell). The solid light blue curve corresponds to the loss of our experiment. The dashed green line shows the delay in free space. The Rabi frequency along *z* is displayed in the top inset for both f.sp (dashed) and LC (solid). For comparison, the delay of an improved LC^40^ with reduced loss *α*_LC_/100 and decoherence rate *γ*_d_ = 100 kHz is shown as a dark blue curve. Rabi frequency along *z* in this improved LC (bottom inset, solid line) and corresponding f.sp. (bottom inset, dashed line) are shown as well

As one of the main results of this work, we derived the ground-state decoherence rate *γ*_d_ from measurements of EIT in free space and in the LC from the heuristic approach used before to gain further insight in the influence of the LC on coherence properties. The depth of the transparency window depends on *γ*_d_, a parameter that is related to the temperature of the vapour, and the co-linear alignment of the light fields in the experiment. To avoid spurious results of *γ*_d_ from the fittings, the temperature remains constant through-out each series of varying powers and the beams keep the same alignment over the whole experiment. This gives a solid comparison between the free-space and the LC decoherence rates *γ*_d_ values, shown in Fig. 4a. The ground-state decoherence rate remains nearly constant *γ*_d_ ≤ 20 MHz in the free-space measurement. For the three different oven temperatures in the LC, *γ*_d_ increases with increasing coupling laser power up to *γ*_0_ = 130 MHz. The effect is more pronounced for a higher oven temperature as expected. Surprisingly, above *P*_c_ = 1 mW the decoherence rate *γ*_d_ decreases again and returns to the free-space level at *P*_c_ = 10 mW. Strong polarisation of the atoms at high intensities could be a reason for a reduction of spin exchange upon inter-atomic and atom-wall collisions. This behaviour is very promising for applications of the LC as integrated devices for quantum storage or delay. To this point, the mechanism behind the decrease has to be further explored with different LC structures.

In order to investigate the concept of a LC as quantum-storage or quantum-synchronisation device^49^, we use the measured EIT parameters to evaluate possible delays of light pulses. The group velocity of a frequency component of a wave packet

1 $$v_g(\nu )/c_0 = \left( {n(\nu ) + \nu \frac{{\partial n}}{{\partial \nu }}} \right)^{ - 1}$$

depends on the refractive index of the dispersive medium n(*ν*), its derivative, and the vacuum speed of light *c*_0_. Therefore, in a medium with a strongly varying susceptibility, such as atomic resonances with EIT, a considerable delay of a light pulse can be anticipated^50^. To evaluate the performance of the cell-integrated LC as device for delay of light, propagation of a Gaussian pulse through the LC in the vapour cell is numerically simulated using the transfer function (Supplementary Section IV).

In the calculation, the light pulse (orange line) is sent through the LC and compared with the free-space propagation scenario, using our experimental parameters of ground decoherence and maximum Rabi frequency for a vapour at 80 °C, as shown in Fig. 4b. The pulses in both cases preserve their shape with a reduction of their group velocity to almost a thousandth of the speed of light in vacuum (Supplementary Section IV). The well-established fractional delay^51^ multiplied by the efficiency of the pulse propagation, giving the figure of merit *F*, also indicates that both cases perform almost identically (Table 1). The effective beam intensity is higher in the LC than in free space due to longer propagation at the focal beam diameter. The LC, therefore, provides a higher effective Rabi frequency and wider EIT window (Fig. 3b). An additional figure of merit can be given as the integrated on-axis intensity over the propagation length, $\tilde I$. In the LC $\tilde I$ is four times the value compared to free-space (Table 1), showing a substantial enhancement of intensity in the structure.

**Table 1:** Comparative overview on light delay in different types of waveguides

		F. sp.	LC	HC PCF^29^	Rectang. ARROW^52^	HC PCF^20^	Improv. LC
${\tilde{I}}_{{\mathrm{str}}}/{\tilde{I}}_{({\mathrm{f}}{\mathrm{.sp}})}$ maximum		1 1	4.4 19	– –	– 130	– 175	75 84
Length (mm)		5	4.5	200	4	200	19.5
Delay *t*_D_ (ns)		3.9	3.1	–	16	12.5	29
Frac. delay *F*		0.055	0.063	–	0.2	30	0.89†
Fill time (days)		~1	~1	~10^4^	–	~10^4^	~1
Chip-integrable		No	Yes	No	Yes	No	Yes
LIAD required		No	No	Yes	No	Yes	No
$\varrho_{{\rm{atom}}}$ (10^10^ cm^−3^)		10	7	76	120	5	~7*

Figure-of-merit parameters, such as the intensity enhancement in the corresponding structure $\tilde I_{{\mathrm{str}}}/\tilde I_{({\mathrm{f}}.{\mathrm{sp}})}$ and its maximal achievable value assuming no losses (max), are displayed in the first row. The improved LC offers the best overall package with its fast filling, high intensity enhancement, large fractional delay, and longest expected delay compared to the other structures, while still maintaining a small footprint for chip-integrability. The comparably moderate fractional delay † and vapour density ∗ are imposed on the LC only because the present experimental conditions were assumed for extrapolation. They can still be improved by application of Raman-based memory^20^ (for †) and increasing the vapour temperature or implementation of LIAD (for ∗), respectively. See ‘Discussion’ in the main text for details

The behaviour of the current LC can be drastically improved by the use of a dual-ring structure which was recently realised^40^. In order to show this, we performed a calculation, where we assumed an increased length of 19.5 mm and low ground-state decoherence of 100 kHz, this would even allow for a 100-fold reduction in modal attenuation^40^ (see Supplementary Section IV). The larger interaction length will allow for higher optical densities when the atom density is limited and, thus, provide longer delay times for the application of a photon delay. The strongly reduced loss of *α*_LC_ = 0.05 dB mm^−1^ in the improved light cage leads to an almost constant intensity along the light cage. We can therefore reduce the power of the coupling laser to set a smaller but constant Rabi frequency in the improved light cage to achieve the same EIT window width as in the experiment (Fig. 4b, bottom inset). We found that a low-loss LC improves the EIT splitting more than 20-fold as compared to a strongly focused beam in free-space (Table 1). This would result in a group velocity closer to the free-space situation, yielding a longer pulse delay (dark blue line) due to the greater length of the structure. The lower attenuation also gives an increased efficiency, pushing the figure of merit *F* one order of magnitude compared to the current LC. Therefore, reduction of loss, as predicted for multi-layer LCs^40^, is the most important optimisation parameter for a LC structure. As depicted in the lower inset in Fig. 4, it would clearly resemble a rod of light with nearly constant intensity where atoms can coherently interact over lengths of centimetres.

## Discussion

As a final point of this work, we discuss the properties of the LC in relation to both free-space guiding and other hollow-core waveguides in Table 1. All presented structures are comparable to the LC in terms of one target application; light delay. The parameters listed in Table 1 have been chosen to quantify and describe each device’s potential to perform as a vapour-based light-delay device. One can readily notice the excellent filling time of the LC as compared to its competitors.

Moreover, it does not require LIAD to produce adequate vapour densities, which represents a substantial advantage on other HC PCFs. In future experiments, the use of a double-ring LC will considerably increase the intensity enhancement $\tilde I/\tilde I_{({\mathrm{f}}.{\mathrm{sp}})}$ as well as the fractional delay, quantified by the figure of merit *F*. The maximal en hancement agrees with observations by other sources^20,52^. To simulate the behaviour of the double-ring LC, the present experimental results were used as a basis for extrapolation. On-resonant EIT light delay limits the maximum transmission bandwidth and, therefore, the fractional delay. Broader bandwidths and larger fractional delay († in Table 1) can be reached by implementation of Raman-memory schemes^20^. Furthermore, higher atomic densities (∗ in Table 1) are achievable with higher vapour temperature (>80 °C) or via LIAD. The potential of the LC as an integrable, vapour-based light-delay device, that surpasses other integrated platforms prone to the limitations (i) to (iii) from the Introduction, is clearly highlighted by the overview given in Table 1.

The large range of applied optical powers from 10 µW to 25 mW lays the foundation for nonlinear experiments in the light cage with optical control over multiple orders of magnitude. Note that the fundamental loss rate of *α*_LC_ < 0.1 dB mm^−1^ for the fundamental mode of the light cage sets the limit of reasonable light-cage lengths to a few tens of millimetres, which is sufficient for many applications as for instance shown in this work. A new, reasonable maximum length of the light cage of 3 cm was presented in a recent work^42^. The flexibility and versatility of designs achievable with the fabrication method are both undeniable advantages when it comes to interfacing LCs with fibre optics or planar photonics. For instance, interfaces could be realised by printing planar waveguides or fibre mounts directly at the in- and outputs of the LC with better than micrometre precision. In particular, combined with an optical-fibre–glass through-put^53^ into the vapour (or liquid^54^) cell, one could easily envision a novel class of integrated chip devices in networks with a small volume of 1 mm × 0.5 mm × 20 mm. Ultimately, this would allow for the realisation of a chip-scale vapour cell made entirely by direct laser writing. The flexibility of 3D nanoprinting allows for customised adaptation of the strand array beyond the hexagonal arrangement used here in order to, e.g., maximise the coupling efficiency from light cage to an external fibre. Moreover, this implementation approach enables sophisticated studies such as exploring the potential of the light cage geometry for reducing the geometric footprint of the light cage while maintaining a large fraction of the optical power inside the LC core (see Supplementary Fig. S4). Delay of light in much shorter light cages could be implemented for fully chip-integrated quantum networks by increase of the optical density via the vapour temperature and smaller group velocities in narrower EIT windows for spectrally narrow light sources. Further studies will investigate the resilience of the alumina-coated light cage in an alkali-atom environment as alkali vapours, especially at elevated temperatures, are chemically highly reactive. The effect of the light cage on coherent properties of the light-matter interaction will be subject to continued investigation in light cages with different distances of the mode to the structure surfaces by comparison of various core diameters (see Supplementary Fig. S4).

In conclusion, we have observed electromagnetically-induced transparency (EIT), a prominent quantum-optical effect, of atoms interacting coherently with a non-diffractive beam of light confined inside a novel on-chip hollow-core light cage (LC). We found a potential interaction length of centimetres, a high density of Cs atoms in the interaction zone, and a remarkably low decoherence rate *γ*_d_ in combination with versatility and stability of the structure integrated inside a room-temperature vapour cell. Further improvements of the structure, such as insertion of a buffer gas, adding another ring to the LC or direct fibre coupling are straightforward. Generation of broad EIT windows with expected light delay in the order of *c*_0_/1,000 lays an excellent foundation to synchronise photon arrival in quantum networks or to realise compact quantum storage on-a-chip. Our results represent a major step forward by an unprecedented hybrid integration of designer laser-written structures and atom cells.

## Materials and methods

### Experimental design

#### Light cage: fabrication and integration into alkali vapour cell

The LC in this work was printed by 3D two-photon polymerisation lithography (TPL) on a silicon substrate. A commercially available 3D-printing system (Photonic Professional GT, Nanoscribe GmbH) was used with a negative-tone photosensitive resist (IP-Dip, Nanoscribe GmbH). We used a ×63 objective with NA = 1.4 to achieve sub-micrometre lateral resolution of approximately 400 nm. Instead of importing a CAD file which is widely used in 3D printing, we used a custom-made general writing language (GWL) program. This allows for optimisation of the writing conditions of different parts comprising the LC for mechanical stability. The lateral position of the laser beam was controlled by galvometer mirrors at a speed of 55 mm s^-1^. The system is equipped with a *λ* = 780 nm NIR-femtosecond laser with pulse width of 100 fs and a repetition rate of 80 MHz. The mean power of the beam before entering the objective was 12.4 mW. The distance between each horizontal layer was 150 nm, the distance between lines within a horizontal layer was 100 nm. After printing, we used low-temperature ALD to coat the structure with alumina at a growth rate of 2.2 Å cycle^−1^ at 30 °C. The chip is placed on an aluminium mount and held tightly with a glass clamp that was melted to the main glass body. The glass cell was custom-made with optical-quality glass for beam access. The cell was filled with 1 g of caesium by breaking a glass ampoule under high vacuum. It is placed in an oven with resistance heaters and isolated from the environment by a polyoxymethylene (POM) housing.

#### EIT setup

In the EIT experiment, displayed in Fig. 2a, probe light (EYP-DFB-0894, eagleyard Photonics) and coupling light (DL 100 and BoosTA, Toptica) are each collimated (60FC-F-4-A8-07, Schäfter + Kirchhoff) and superimposed on the polarising beam splitter PBS. The beams are matched to the light-cage mode by lens L1 and re-collimated afterwards at L2 (both A220TM-B, Thorlabs). In the case of free-space EIT, the lenses L1 and L2 are confocally aligned, resulting in a focal beam waist *w*_0_ = 4.6 μm and a Rayleigh length of *z*_R_ = 76 μm. The coupling field is linearly polarised at the first linear polariser LP1. Orthogonal orientation of the second linear polariser LP2 and optimisation of half-wave plate HWP and quarter-wave plate QWP accomplish suppression of the coupling laser intensity of the order of −60 dB. It is limited by the extinction ratio of the polarisers and due to polarisation rotations by passing through the cell windows with focused beams and the atomic medium. An iris is used to spatially filter scattered probe and coupling light before QWP. The transmitting probe light is collected into a single-mode fibre (60FC-F-4-A8-07, Schäfter + Kirchhoff) and detected by a photodiode PD.

## Supplementary information

Supplementary Materials

## Data Availability

The data that support the plots within this paper and other findings of this study are available from the corresponding author upon reasonable request.

## Code availability

The code that supports the plots within this paper and other findings of this study is available from the corresponding author upon reasonable request.
