# Publisher Correction: Coherent interaction of atoms with a beam of light confined in a light cage

**DOI:** 10.1038/s41377-021-00571-0

**Published:** 2021-07-01

**Authors:** Flavie Davidson-Marquis, Julian Gargiulo, Esteban Gómez-López, Bumjoon Jang, Tim Kroh, Chris Müller, Mario Ziegler, Stefan A. Maier, Harald Kübler, Markus A. Schmidt, Oliver Benson

**Affiliations:** 1 Department of Physics and IRIS Adlershof, Humboldt-Universität zu Berlin, 12489 Berlin, Germany; 2 Chair in Hybrid Nanosystems, Nanoinstitute Munich, Faculty of Physics, Ludwig-Maximilians-Universität München, 80539 Munich, Germany; 3 Department of Fiber Photonics, Leibniz Institute of Photonic Technology, 07745 Jena, Germany; 4 Competence Center for Micro- and Nanotechnologies, Leibniz Institute of Photonic Technology Jena, 07745 Jena, Germany; 5 Department of Physics, Imperial College London, London, SW7 2AZ UK; 6 Physikalisches Institut and Center for Integrated Quantum Science and Technology, University of Stuttgart, Pfaffenwaldring 57, 70569 Stuttgart, Germany; 7 Otto Schott Institute of Material Research, 07743 Jena, Germany

**Keywords:** Integrated optics, Atom optics, Polymers

Correction to: *Light: Science & Applications*

10.1038/s41377-021-00556-z published online 31 May 2021

After publication of this article^1^, it is noticed the article contained an error.

In Table 1, the data in the line ‘Length (mm)’ is missing. The complete Table 1 is provided in this correction.

**Table 1:** Comparative overview on light delay in different types of waveguides

	F. sp.	LC	HC PCF^29^	Rectang. ARROW^52^	HC PCF^20^	Improv. LC
$\widetilde {\mathrm{I}}_{{\mathrm{str}}}{\mathrm{/}}\widetilde {\mathrm{I}}_{\left( {{\mathrm{f}}.{\mathrm{sp}}} \right)}$	1	4.4	–	–	–	75
maximum	1	19	–	130	175	84
Length (mm)	5	4.5	200	4	200	19.5
Delay t_D_ (ns)	3.9	3.1	–	16	12.5	29
Frac. delay F	0.055	0.063	–	0.2	30	0.89†
Fill time (days)	~1	~1	~10^4^	–	~10^4^	~1
Chip-integrable	No	Yes	No	Yes	No	Yes
LIAD required	No	No	Yes	No	Yes	No
$Q_{\rm{atom}}$ (10^10^ cm^−3^)	10	7	76	120	5	~7*

The original article has been updated.
