# Supplementary material for: Coherent interaction of atoms with a beam of light confined in a light cage
Source: Light Sci Appl. 2021 May 31;10:114. doi: 10.1038/s41377-021-00556-z (PMC8166889; doi:10.1038/s41377-021-00556-z)
Supplement: Supplementary file 1 — Supplementary Materials [file 41377_2021_556_MOESM1_ESM.pdf]

Supplementary Materials for

Coherent interaction of atoms with a beam of light confined in a light cage

Flavie Davidson-Marquis,[1†] Julian Gargiulo,[2†] Esteban Gómez-López,[1†] Bumjoon Jang,[3†] Tim Kroh,[1†]* Chris Müller,[1] Mario Ziegler,[4] Stefan A. Maier,[2,5] Harald Kübler,[6] Markus A. Schmidt,[3,7] & Oliver Benson[1]

[1] Department of Physics and IRIS Adlershof, Humboldt-Universität zu Berlin, 12489 Berlin, Germany.

[2] Chair in Hybrid Nanosystems, Nanoinstitute Munich, Faculty of Physics, Ludwig-Maximilians-Universität München, 80539 Munich, Germany.

[3] Department of Fiber Photonics, Leibniz Institute of Photonic Technology, 07745 Jena, Germany.

[4] Competence Center for Micro- and Nanotechnologies, Leibniz Institute of Photonic Technology Jena, 07745 Jena, Germany.

[5] Department of Physics, Imperial College London, London SW7 2AZ, UK.

[6] 5. Physikalisches Institut and Center for Integrated Quantum Science and Technology, University of Stuttgart, Pfaffenwaldring 57, 70569 Stuttgart, Germany.

[7] Otto Schott Institute of Material Research, 07743 Jena, Germany.

† These authors contributed equally to this work.
* Corresponding author: tim.kroh@physik.hu-berlin.de

SUPPLEMENTARY MATERIALS

I. Light cage design & sample fabrication

Light cage concept.

The light-cage structure used in this work and its properties are shown in Article Figure 1. Twelve parallel polymer strands (diameter D_s = 3.6 μm) are arranged in a hexagonal lattice (lattice constant Λ = 7 μm), encircling a hollow core in the centre[1] (Article Figure 1c, d). The structure tightly confines and guides the light in the 12.6 μm wide core mode over a length of 4.5 mm by the antiresonant effect[2] and via inhibited coupling[3]. Specifically, the individual modes in the strands (cf. Article Figure 1f) produce hybridised supermodes which are anti-resonant with the central core

mode within specific spectral domains leading to a sequence of high transmission bands (Article Figure 1b). Note that due to the absence of total internal reflection the formed leaky modes radially dissipate energy while propagating[4], with most of their fields concentrated in the hollow core (Article Figure 1g). According to simulations, the LC concept reaches up to 99.99 % of power in the core (Article Figure 1e). The interaction length between light and core medium is determined by the length of the LC, which is 4.5 mm in the presented case. This provides 70 times longer interaction at high intensity in comparison with the Rayleigh length of a focused beam with identical beam waist. One notable feature of LC is the side-wise openness allowing the surrounding low-pressure vapour to enter the core and efficiently interact with the light field. A representative comparison of filling times between a capillary-type hollow-core structure and the LC is shown in Article Figure 1h. More than two orders of magnitude faster filling makes the LC concept a beneficial platform, in particular for vapour-based applications.

Sample fabrication.

We fabricated LCs on a silicon substrate by 3D nanoprinting with a two-photon polymerisation lithography system (Nanoscribe GT, details in Methods section). The waveguide design parameters (i.e. D_s and Λ, top inset in Article Figure 1a) are chosen according to finite element simulations, followed by experimental prototyping to simultaneously realise strong mechanical stability, efficient light guidance and an as-large-as-possible openness. Combining the alkali resistance, obtained by film coating, with the intrinsic properties of the LC – such as strong light confinement, low optical loss, long-term stability, and the virtually instantaneous filling of room-temperature alkali vapour – one obtains a particularly attractive novel class of integrated quantum devices for experiments focusing on strong coherent light-matter interaction.

Light cage: Durability

The LC with alumina coating proves stable against the chemically highly-reactive Cs atmosphere. The transmission spectrum in Article Figure 1b was measured ten months after the LC was placed into the cell. Light fields have been guided in the LC with intensities of the coupling laser field up to the order of $I_c = 20$ kW cm^{-2} (at $P_c = 30$ mW). More than 99 % of the field in the centre mode of the LC are in a distance to the confining strands of about 6 μm. Thus, only marginal interaction of the intense fields with the LC material is expected. A more thorough examination of the optical damage threshold of the LC material and structure will be required for nonlinear optical experiments which strive for high intensities in order to achieve near unity efficiencies.

II. Gas diffusion

The diffusion of atoms, for instance Rb, in a glass capillary does not follow classical, or modified, Knudsen models for transport[5], since presumably atoms may stick on surfaces of the underlying waveguide structure. In Ref. 6, the authors show that the atoms stick to the wall for times on the order of microseconds and speculate that this results from a little diffusion of the atoms into the glass. The transport time t_t, displayed in Article Figure 1h, in glass capillaries (time between initial Rb loading and the first instance of > 2 % absorption on the unloaded side of the testing platform) is given by Ref. 5 as $t_t = \zeta\,(L/d^2) + t_{stable}$ (L: length of capillary, d: diameter of capillary, ζ: slope, t_{stable}: time required for wall–vapour interaction to stabilise) in the reservoirs on both sides of the capillary. The parameter $\zeta = 24$ μm days for an uncoated sample at $T = 90$ °C was taken from Ref. 5. For the purpose of comparison, the structure diameter is set to $d = 20$ μm in order to approximate all discussed types of waveguides. Based on the quick availability of absorptive spectra in the light cage after filling, the stabilisation time is chosen at the order of $t_{stable} = 1$ day.

The representative length L is then assumed to be the length of the waveguide in case of the closed ARROW or fibre structures. In the open-wall LC, on the other hand, L is assumed to be the total structure diameter, since the atoms can flow through the LC from all sides (see highlighted areas in Article Figure 1h). Filling of the LC takes three orders of magnitude less time than centimetres-long capillary-type geometries where the filling length is given by the length of the waveguide. Due to the sidewise accessible core. Between experiments, even faster stabilisation times of less than a day were observed, which is probably due to the geometry of the LC where atoms flow through from multiple directions and not only in a linear manner as in the other structures.

The atom density N/V inside the LC was inferred from reference transmission spectra that were taken without a coupling field. At an identical oven temperature (T_{oven} = 80 °C) the atom density inside the LC, $(N/V)_{LC} = (6.9 \pm 0.3)\cdot 10^{10}$ cm^{-3}, is less than a factor of two smaller than 3 mm above the LC in free space: $(N/V)_{f.sp} = (1.02 \pm 0.02)\cdot 10^{11}$ cm^{-3}. This is, however, a remarkably small reduction as compared to experiments in hollow-core fibres, where additional means such as light induced atomic desorption (LIAD) are essential to provide an acceptable vapour density[7].

Compared to other hollow-core waveguide structures, the LC uniquely provides a reduced presence of adherable surfaces, drastically improves the filling efficiency with low-pressure vapour, and minimises the atom-wall collisions, which otherwise induce decoherence[8]. In particular, no additional desorption mechanism is necessary to reach sufficiently high atom densities, which prevents quantum channels with signals at the single-photon level to be filled with numerous high-power laser fields and facilitates filtering.

III. Setup: Efficiencies and losses

The setup transmission loss with the light cage was measured to be $\eta_{setup,LC} = \eta_{1,LC} + \eta_2 + \eta_{3,LC} = -34.9$ dB. The overall light-cage loss $\eta_{1,LC} = -30.1$ dB was measured from just before lens L1 to behind an iris, placed right after lens L2, for a beam that is non-resonant with the caesium (Cs) atoms. See Supplementary Figure S1 for visualisation. Pulse-propagation simulations through the light cage (LC) suggest a loss rate of $\alpha_{LC} = 5.0$ dB mm^{-1} to reproduce the susceptibility profiles comparable to the measured spectra. In particular, the presence of the shoulder-like features can only be reproduced with this relatively high loss coefficient (see Supplementary Section IV Analysis). Reflections at the cell-glas surfaces and the lenses cause a loss of $\eta_{refl} = -1.3$ dB. It is impossible with the present methods to attribute the residual loss of $\eta_{res} = -6.3$ dB $= \eta_{1,LC} - \alpha_{LC} L - \eta_{refl}$ to a certain origin, neither loss at a specific scatterer or defect along the light-cage structure nor at the front or output facet. $\eta_2 = -2.1$ dB is measured between the locations after lens L2 and behind LP2 to account for losses in the polarisation elements. These losses arise from depolarisation in the LC. Finally, $\eta_{3,LC} = -2.7$ dB is the measured coupling efficiency into the single-mode fibre (SMF). In the case of the free-space experiment we find $\eta_{setup,fs} = \eta_{1,fs} + \eta_2 + \eta_{3,fs} = -4.95$ dB that are composed off $\eta_{1,fs} = \eta_{refl} = -1.3$ dB, $\eta_2 = -2.1$ dB being identical for the LC and the free-space experiment, and $\eta_{3,fs} = -1.55$ dB. The difference $\eta_{3,LC} > \eta_{3,fs}$ leads to the conclusion that the propagation of the probe field in the recent light cage affects the spatial shape of the mode. In the normalised spectra (see Supplementary Material Section IV Analysis) the EIT transmission goes as high as 99 % on resonance, for the highest powers, at any of the measured oven temperatures. This implies that η_1 would remain unaltered in the high coupling-power regime.

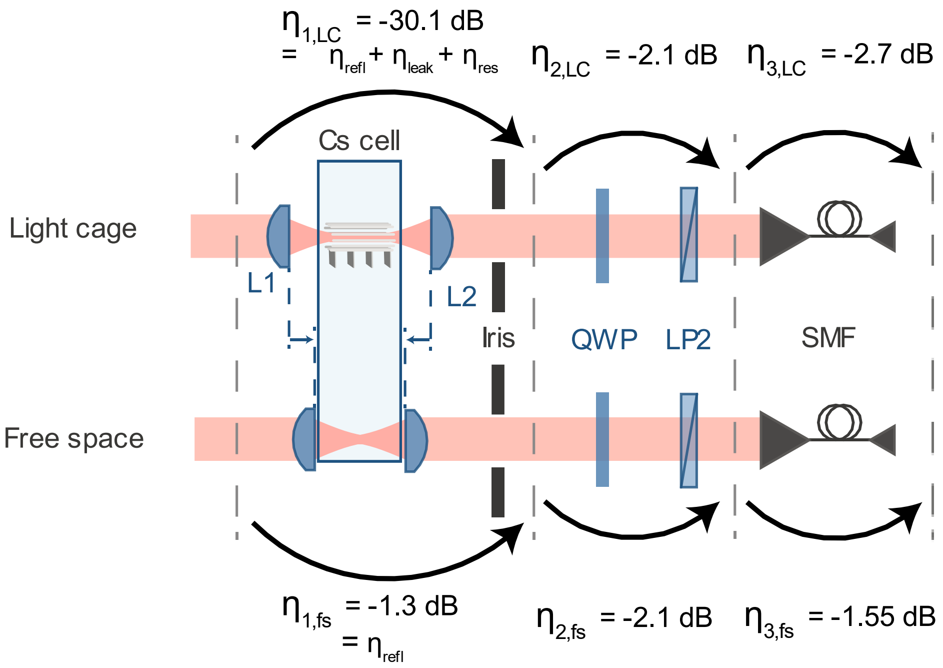

Fig. S1. Comparison of the losses for the light-cage and free-space experiments. The overall losses at the vapour cell $\eta_{1,LC}$ are composed of the reflections at glass surfaces η_{refl}, the loss rate of the leaky mode $\eta_{leak} = -\alpha_{LC} L$ along the light cage, and the residual, unidentified loss η_{res}. Only the reflection losses $\eta_{refl} = \eta_{1,fs}$ are present in the free-space experiment. The probe-beam loss of $\eta_2 = -2.1$ dB are suspected to originate from passing the cell windows with strongly focused, polarised beams and the necessary polarisation filtering of the coupling-laser light. The fibre-coupling losses are $\eta_{3,LC} = -2.7$ dB and $\eta_{3,fs} = -1.55$ dB, respectively.

To confirm proper beam coupling into the LC (cf. mode profile in Article Figure 1g), the transmission spectrum at near-infrared wavelengths was measured by in-coupling of broadband light (SuperK, NKT Photonics). The obtained spectrum, normalised to the transmission in absence of the LC, clearly shows the characteristic spectral periodicity of anti-resonant structures as well as an overall transmission reduction towards longer wavelengths[9,10], thus certifying LC-guided light.

IV. Analysis

Data processing

Normalisation of the data has been performed to compensate for the power changes when scanning the probe diode laser. Three data sets were taken for each scan over 20 GHz: (1) the measurement of the EIT spectrum with probe and coupling light on, (2) a simple spectrum recorded with a probe scan while the coupling laser is blocked, and (3) the constant background signal given by the coupling light. The third was only detectable over the detection noise for the highest coupling laser powers in the range of tens of milliwatts, but was measured every time for completeness. For further analysis of the data, the background (3) was subtracted from (1) and normalised with a fourth-order polynomial fit to the behaviour of scan (2) far away from (and between the two pairs of) the resonances of the caesium D1 transition.

Transmission and absorption fitting.

The transmission spectrum can be explained by means of the transfer function of the medium under the presence of EIT. For an optically dense medium this is given as $T = \exp[ik\, n_c\, z]$, where k is the wavenumber, z the propagation length and n_c the complex refractive index, $n_c(\delta_p) = \sqrt{1+\chi(\delta_p)}$.

Here, χ is the electric susceptibility of the atomic vapor in the linear regime, that can be calculated in presence of EIT as in Ref. 11 as

$$\chi(\delta_p) = \frac{|\mu_{31}|^2 \rho_N}{\varepsilon_0 \hbar}\left[\frac{4\Delta(\Omega_c^2 - 4\Delta^2 - \gamma_d^2)}{|\Omega_c^2 + (\gamma_{31} + i2\Delta)(\gamma_d + i2\Delta)|^2} + i\frac{8\Delta^2\gamma_{31} + 2\gamma_d(\Omega_c^2 + \gamma_{31}\gamma_d)}{|\Omega_c^2 + (\gamma_{31} + i2\Delta)(\gamma_d + i2\Delta)|^2}\right] \quad \text{(S1)}$$

for the probe frequency detuning $\Delta = 2\pi\, \delta_p$ (Article Figure 1a, inset) with the probe-beam transition dipole moment μ_{31}, the atom number density ρ_N, the vacuum permittivity ε_0, Planck's constant $\hbar$,

the coupling field Rabi frequency Ω_c, as well as the ground-state and excited-state decoherences, γ_d and $\gamma_{31} = \Gamma_3 + \gamma_{3d}$, respectively. γ_{3d} accounts for additional homogeneous broadening induced by the probe-beam power.

The results shown in Article Figure 2b and, here, in Supplementary Figure S3 were fitted using $\chi(\delta_p)$ as starting function from where the transmission is calculated. The spectral lines are homogeneously and inhomogeneously broadened due to the high-intensity probe beam and the thermal drift of the atoms respectively. The corresponding widths of each broadening mechanism are left as free parameters to account for the reduction of atomic density in the waveguide. The Rabi frequency and the decoherence rate between the two ground states are obtained from the fits.

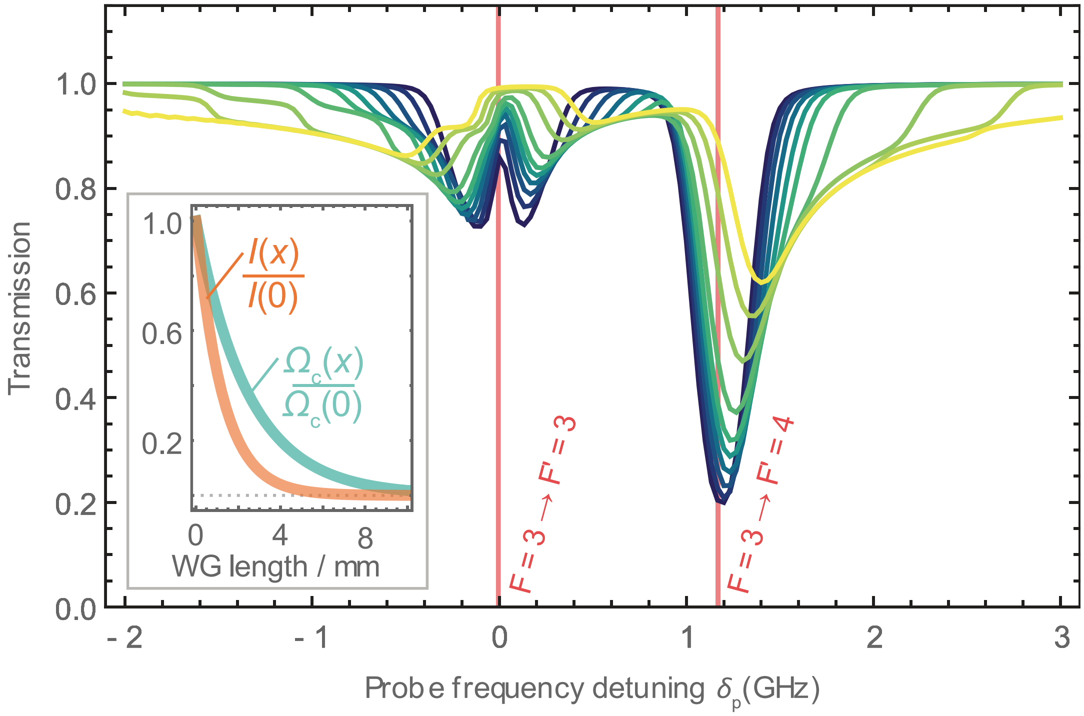

Fig. S2. Simulation of EIT in a lossy hollow-core light cage. Over the length of the LC, mode losses α_{LC} lead to a decay of the intensity or power of the involved fields $I(x)/I_0 = 10^{-\alpha_{LC}\cdot x/10\mathrm{dB}}$ (inset, orange). Accordingly, the coupling Rabi frequency decreases as $\Omega_c(x)/\Omega_c(0) = 10^{-1/2\alpha_{LC}\cdot x/10\mathrm{dB}}$ (inset, blue). The displayed EIT spectra are calculated by subdividing the 4.5 mm LC in 40 equal segments. In each cell a constant, but from cell to cell decreasing, Rabi frequency is assumed. The different curves correspond to $\Omega_c(0)$ from 0.6 GHz (dark blue) to 5 GHz (yellow) at the entrance facet.

These deviations arise from a Rabi frequency $\Omega(\delta_p, z)$ that varies with the propagation distance z. In free space, both beams are tightly focused Gaussian beams, producing, at first, a rapidly increasing and then, after the focal point, a strongly decreasing field amplitude along the z-axis, as shown in Article Figure 4b insets. This changes the transmission of the medium at every point along the propagation axis. To simulate the transmission presented in Supplementary Figure S2, the experimental conditions are used. The Rabi frequency $\Omega_c(\delta_p,z)$ is calculated employing a Gaussian beam tightly focused by a lens of 11 mm focal length, giving a 4.6 μm beam waist in free-space. Under such conditions, on the optical axis the Rabi frequency is $\Omega(z) = \Omega_0\, w_0/w(z)$, where Ω_0 and w_0 are the maximum Rabi frequency and beam waist at focus, respectively, and $w(z)$ is the 1/e radius at a distance z. Meanwhile, the LC case is simulated by a Gaussian beam focusing into the facet for 0.25 mm (distance in the vapour cell between optical window and the LC facet), then an exponential loss approximated as $I(z)/I_0 = 10^{-\alpha_{\mathrm{LC}} z/10\mathrm{dB}}$ with experimentally determined modal attenuation of $\alpha_{\mathrm{LC}} = 5.0$ dB mm^{-1} through the 4.5 mm long LC, followed by additional 0.25 mm of diverging beam (from the facet to the cell wall). The maximum Rabi frequency $\Omega_0 = 8$ GHz is estimated to match the Rabi frequency $\tilde{\Omega}_c = 400$ MHz from the measured transmission spectra. Using $\Omega(\delta_p, z)$ in Supplementary Equation (S1) allows to calculate $\chi(\delta_p,z)$ along the propagation axis. The resulting spectrum in Article Figure 3d (main text, solid blue line) is deduced from the integrated susceptibility along the cell length, $\tilde{\chi}(\delta_p) = \int_0^L \chi(\delta_p, z)\mathrm{d}z$, where $L = 5$ mm is the length of the vapor cell containing the LC.

Pulse delay simulations

The propagation of a Gaussian pulse $E_{\mathrm{in}}(t)$ with 14 ns temporal FWHM (Article Figure 4b) is performed via the complex transfer function T using $\tilde{\chi}(\delta_p)$, i.e. $E_{\mathrm{out}}(\delta_p) = T_\mu(\delta_p)E_{\mathrm{in}}(\delta_p)$, obtaining

both absorption and dispersion of the pulse by the atoms in the LC. In order to translate from temporal space to frequency space where the transfer function is calculated, Fourier transforms are used back and forth, $E_{in}(\delta_p) = F\ [E_{in}(t)]$. The extrapolation of the delaying capabilities of the LC (Article Figure 4b) is performed in the same way using a greatly reduced loss for a dual-ring structure[10], this is $\alpha_{LC} = 0.05$ dB mm^{-1}, and an increased length $L = 19.5$ mm, still within the capabilities of the manufacturing process. Low ground decoherence $\gamma_d = 100$ kHz is assumed. Due to the apparition of highly absorbing features near resonance, within the EIT dip, the pulse is shifted 50 MHz to avoid additional absorption and pulse deformation.

Article Figure 4b displays a situation where we assume an atom density inside the LC that corresponds to a free-space, ideal-gas temperature of $T_{vapour} = 80°C$ and an initial Gaussian input pulse with 14 ns FWHM (orange line). The resulting delayed pulses are shown after propagating in free space (dashed green line) and in the LC (light blue line). The decoherence of the F = 4 ground state was taken from a typical experimental value as $\gamma_d = 20$ MHz, while peak Rabi frequencies of 3 GHz and 8 GHz were chosen for LC and free space, respectively, to maintain comparability (top inset in Article Figure 4b). These pulses preserve a similar temporal width after propagation, while the delay is $t_D = 3.9$ ns in free-space and 3.1 ns in the LC, corresponding to respective group velocities of $v_{f.sp} = 4.3\cdot10^{-3}\ c_0$ and $v_{LC} = 5.3\cdot10^{-3}\ c_0$. To extrapolate the capabilities of the LC to the two-layered structure[10] a reduced modal attenuation $\alpha_{LC}/100$ is considered together with an extended LC length of 19.5 mm. An improved ground decoherence $\gamma_d = 100$ kHz is used. In Article Figure 4b (dark blue line), a large increment of the delay is observable in the improved LC, $t_D = 29$ ns ($v_{LC} = 2.3\cdot10^{-3}\ c_0$), for the same input pulse, with minimal spectral broadening. This arises from a nearly constant value of Ω_c across the entire structure due to nearly-negligible modal attenuation (bottom inset in Article Figure 4b).

Integrated on-axis intensity and weighted fractional delay

The EIT windows for the present experimental conditions allow for the incoming pulses to be transmitted with η = 23.3 % (atoms in free space) and 30.5 % (LC) efficiency. Taking modal attenuation of the LC (and changes of the mode diameter in free space) into account, the integrated on-axis intensity over the length L of the Cs cell is expressed as $\tilde{I} = \int_0^L I(z)\mathrm{d}z$. In the single-ring twelve-strand LC, used here, this parameter shows an enhancement with respect to the free-space situation of $\tilde{I}_{\mathrm{LC}} / \tilde{I}_{\mathrm{f.sp}} = 4.4$ at same maximum Rabi frequency. However, for the explicit application of an EIT-based photon delay, the fractional delay[12], defined as the ratio between delay time t and pulse width Δt, is a more appropriate quantity. Here, in order to account for modal attenuation losses in the LC and absorption in the atomic vapour we multiply this ratio by the pulse transmission τ (through the atomic vapour) to define the best figure of merit: $F = t_{\mathrm{D}}/\Delta t \cdot \tau$. Using the simulation results for the present LC used in our experiment, the LC (F_{LC} = 0.063) and free space ($F_{f.sp}$ = 0.055) perform almost identical, as expected from Article Figure 4b.

When considering the improved two-layer LC, the transmission efficiency of the LC is increased to τ = 42.9 %. Meanwhile, in the free-space scenario, it is reduced to only 5.6 %. In the comparatively longer vapour cell, the free-space propagating beam covers a longer distance with a larger diameter – or lower intensity – since the Rayleigh length remains constant. In this case, a narrower EIT window is generated than in the LC since low intensities produce small Rabi frequencies. This eventually pushes the figure of merit to F_{LC} = 0.89 (and $F_{\mathrm{f.sp}}$ = 0.04, respectively) while three orders of magnitude less intensity will be required. In a comparable work with a rectangular hollow-core ARROW waveguide with core dimensions 4.75 μm × 12 μm × 4 mm in Rb vapour[13], the extended fractional delay can be extracted as $F^{13} \approx 0.2$ for delay times $t_{\mathrm{D}} \leq 16$ ns. The transmission of τ = 0.44 causes strong spectral filtering of the pulse and, thus, a significantly broadened pulse width from 20 ns to 32 ns.

V. Additional data and heuristic fitting curve

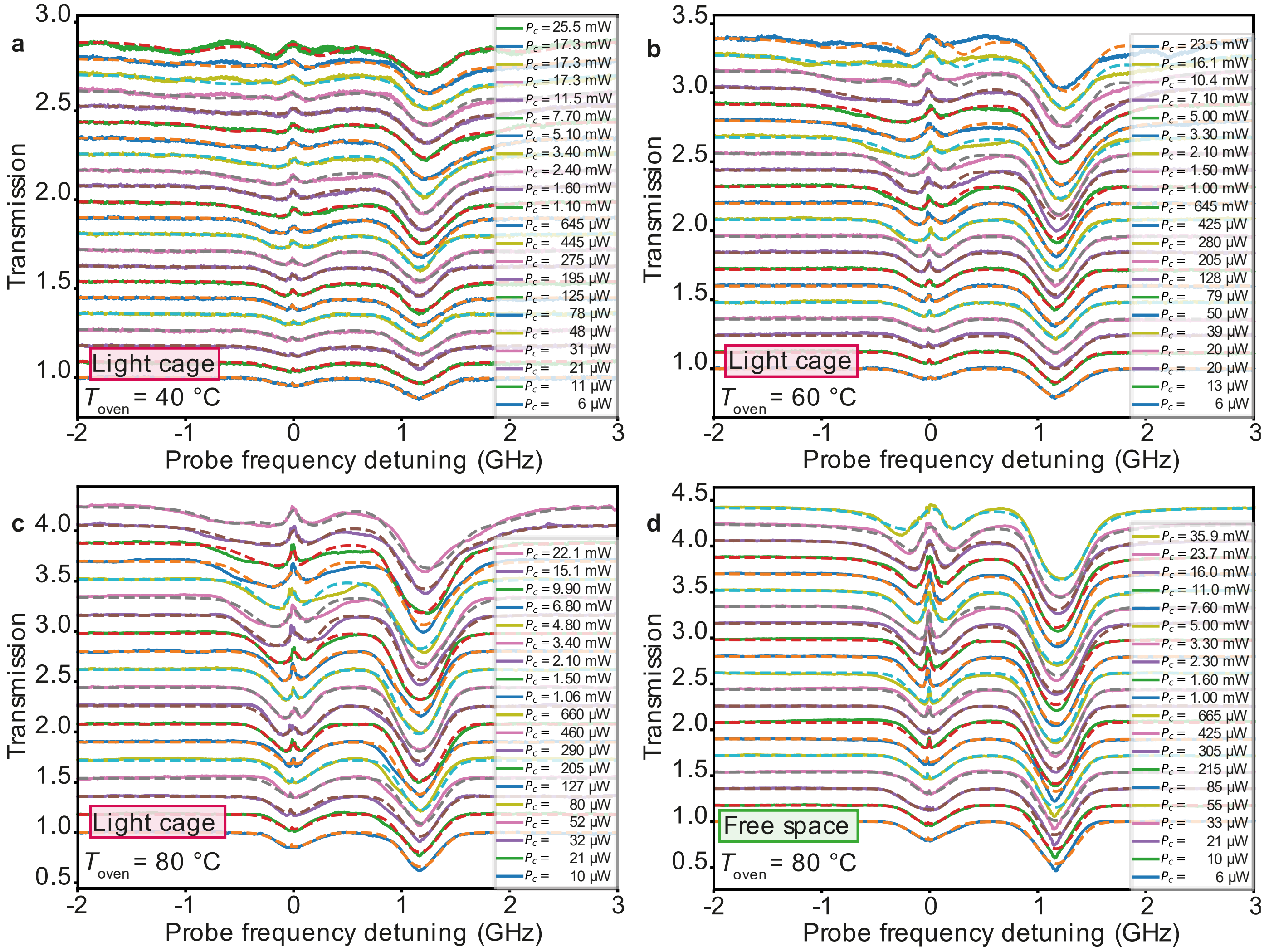

Fig. S3. EIT spectra in the light cage and in free space. The lowest spectrum in each subfigure displays the normalised transmission. The other spectra are shifted with a linear offset for clarity. The dashed lines correspond to the fits performed using the heuristic model.

Here, in Supplementary Figure S3, we present all the measured spectra and their respective fits for three different oven-temperatures of 40 °C, 60 °C, and 80 °C in the LC and 80 °C in free space to compare with. The fits were done using a heuristic approach of virtual resonances. In the experiment, the probe frequency is scanned over a range of 20 GHz around the EIT resonance

(corresponding to a detuning of $\delta_p = 0$), containing the full Cs D1 spectrum, but only the two highest-energetic resonances of the Cs D1 transition are displayed in Article Figure 2b and Supplementary Figure S3. This better represents the details of the EIT line shapes. The appearance of shoulders around the transition where the EIT is located, and its neighbouring resonance, are a sign of varying coupling laser intensity and, hence, changing Rabi frequency along the beam path in the vapour cell. To take this phenomenon into account, a heuristic approach was used. Two virtual resonances are placed at frequencies – δ_v from the centre of the EIT and + δ_v respect to the neighbour peak, giving a good agreement with the measured spectra. The slope of sideband detunings vs. coupling power in the LC (Article Figure 3f) are $\partial\delta_s/\partial P_c$ = 23 to 41 MHz mW^{-1}, which is one order of magnitude larger than in free space ((5 ± 2) MHz mW^{-1}).

VI. Light cage perspectives

Closed cell arrangements can be realised by writing LCs in-between two facing optical fibres, yielding a completely integrated quantum-optical device without any glass windows. Filling can be conducted under an alkali atmosphere or by having a small bowl-like depression for reservoir drops of alkali atoms[14].

From the application perspective, an important aspect is the mechanical and chemical long-term stability of the cell-integrated LCs, since they are exposed to corrosive alkali vapour at different temperatures. The transmission measurement shown in Article Figure 1b was taken about 10 months after the LC was inserted into the Cs-filled cell.

The excellent match of the resonance wavelength (vertical dashed lines in Article Figure 1b) with the spectral locations of the transmission dips clearly reveals that the alumina protection layer sufficiently shields the polymer from being exposed to the alkali vapour. Note, that a similar

transmission measurement showed resonances exactly at the same wavelengths λ_R, together with the diameter tuning slope ($\lambda_R/d \approx 0.5$ nm nm^{-1}) reported in Ref. 15. No degradation of the properties of the polymer as well as the structure of the LC itself was observed. Therefore, the LC concept defines a unique approach for the implementation of integrated complex devices within the context of vapour-based quantum photonics.

To demonstrate the properties of the light cage in the context of light confinement and potential for further on-chip integration, we performed additional simulations revealing the power concentration inside the core region for different transverse extents of the light cage. Specifically, we calculate the fraction of power of the fundamental mode in the core of the light cage at one selected anti-resonant wavelength (i.e., wavelength of smallest loss within one transmission band, here λ = 890 nm) as a function of core radius R (i.e., number of strands N) while leaving all other parameters fixed (d_{strand} = 3.6 µm, Λ = 7 µm, see Supplementary Figure S4). In order to define the core area (and thus the core radius R) as the area lying within a circle passing through the centres of the strands, we have chosen a circular grid (inset of Fig. S4(b)). Please note that this geometry is very similar to the hexagonal light cages used in the experiment due to the large ratio of wavelength and light cage radius and, thus, no significant differences to the experimentally studied structure are to be expected. The vertical dashed blue line in Fig. S4 shows the situation of N = 12, corresponding to the number of strands used in the experiments. $R = \Lambda / (2\cdot\sin[\pi/N])$. As expected, the effective mode index n_{eff} (here relative to unity) decreases for smaller core diameters due to the stronger interaction of mode and strand array (Fig. S4(a)). The corresponding fraction-of-power simulation (Fig. S4(b)) clearly demonstrates the capabilities of the light cage for further reduction of the geometric footprint, showing values greater than 99% for all core radii considered here. Please note that the definition of the core area in the circular light cage geometry becomes increasingly questionable when the number of strands falls below five, which causes us to stop the simulations at N = 6. The

presented results have been achieved for single-ring light cage structures and an even improved performance particularly at small core extents can be anticipated in case the number of rings is increased. Moreover, a variation of the pitch Λ presumably towards smaller values can further increase the fraction of power inside the core, thus overall leaving room for future performance improvement.

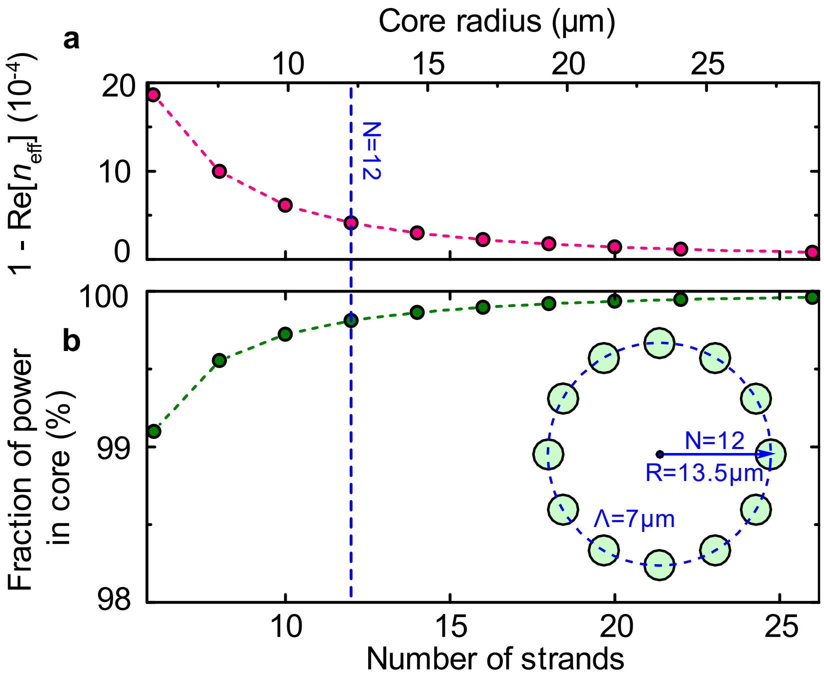

Fig. S4. Power concentration in the LC core in dependency of the LC core radius or number of strands. (a) Relative effective mode index and (b) fraction of power of the fundamental mode in the core of a circular light cage at one selected anti-resonant wavelength (λ = 890 nm) as a function of core radius R (i.e., number of strands N, D_{strand} = 3.6 µm, Λ = 7 µm). The core domain is defined by a circle that passes through the centres of the strands.

It should also be noted that upon improvement of the LC design[10], attenuation rates of 1.5 dB m^{-1} from Ref. 8 will be within reach. In such a low loss regime, the high probe and coupling fields would be virtually constant over the whole length of the LC. This is especially interesting for nonlinear quantum effects in gaseous or liquid[16,17] optical media, where close-to-unity spatial overlap of, e.g., atoms, the single-photon probe, and, simultaneously, the strong coupling fields, are

crucial[18]. Preservation of polarisation in the LC, as another necessary property for nonlinear quantum optics, can be achieved by introducing birefringence through a small asymmetry of the LC geometry.

REFERENCES

1. Jain, C. *et al*. Hollow Core Light Cage: Trapping Light Behind Bars. *ACS Photonics* **6**, 649–658 (2019). URL https://doi.org/10.1021/acsphotonics.8b01428.

2. Litchinitser, N. M., Abeeluck, A. K., Headley, C. & Eggleton, B. J. Antiresonant reflecting photonic crystal optical waveguides. *Optics Letters* **27**, 1592 (2002). URL https://www.osapublishing.org/abstract.cfm?URI=ol-27-18-1592.

3. Debord, B. *et al*. Ultralow transmission loss in inhibited-coupling guiding hollow fibers. *Optica* **4**, 209–217(2017). URL http://www.osapublishing.org/optica/abstract.cfm?URI=optica-4-2-209.

4. Zeisberger, M. & Schmidt, M. A. Analytic model for the complex effective index of the leaky modes of tube-type anti-resonant hollow core fibers. *Scientific Reports* **7**, 11761 (2017). URL https://doi.org/10.1038/s41598-017-12234-5.

5. Giraud-Carrier, M.*et al*. Temperature and wall coating dependence of alkali vapor transport speed in micron-scale capillaries. *Journal of Vacuum Science & Technology A* **35**, 031602 (2017). URL https://avs.scitation.org/doi/10.1116/1.4978888.

6. Sayrin, C. *et al*. Nanophotonic Optical Isolator Controlled by the Internal State of Cold Atoms. *Physical Review X* **5**, 041036 (2015). URL https://link.aps.org/doi/10.1103/PhysRevX.5.041036.

7. Kaczmarek, K. T. *et al*. Ultrahigh and persistent optical depths of cesium in Kagomé-type hollow-core photonic crystal fibers. *Optics Letters* **40**, 5582–5585 (2015). URL https://www.osapublishing.org/ol/abstract.cfm?uri=ol-40-23-5582.

8. Sprague, M. R. *et al*. Broadband single-photon-level memory in a hollow-core photonic crystal fibre. *Nature Photonics* **8**, 287–291 (2014). URL https://www.nature.com/articles/nphoton.2014.45.

9. Jain, C. *et al*. Hollow Core Light Cage: Trapping Light Behind Bars. *ACS Photonics* **6**, 649–658 (2019). URL https://doi.org/10.1021/acsphotonics.8b01428.

10. Jang, B. *et al*. Light guidance in photonic band gap guiding dual-ring light cages implemented by direct laser writing. *Optics Letters* **44**, 4016–4019 (2019). URL https://www.osapublishing.org/ol/abstract.cfm?uri=ol-44-16-4016.

11. Fleischhauer, M., Imamoglu, A. & Marangos, J. P. Electromagnetically induced transparency: Optics in coherent media. *Reviews of Modern Physics* **77**, 633–673 (2005). URL https://link.aps.org/doi/10.1103/RevModPhys.77.633.

12. Akopian, N., Wang, L., Rastelli, A., Schmidt, O. G. & Zwiller, V. Hybrid semiconductor-atomic interface: slowing down single photons from a quantum dot. *Nature Photonics* **5**, 230–233 (2011). URL https://www.nature.com/articles/nphoton.2011.16.

13. Wu, B. *et al*. Slow light on a chip via atomic quantum state control. *Nature Photonics* **4**, 776–779 (2010). URL https://www.nature.com/articles/nphoton.2010.211.

14. Yang, W. *et al*. Atomic spectroscopy on a chip. *Nature Photonics* **1**, 331–335 (2007). URL https://www.nature.com/articles/nphoton.2007.74.

15. Jang, B. *et al*. Fine-tuning of the optical properties of hollow-core light cages using dielectric nanofilms. *Optics Letters* **45**, 196–199 (2020). URL https://www.osapublishing.org/ol/abstract.cfm?uri=ol-45-1-196.

16. Assanto, G., Fratalocchi, A. & Peccianti, M. Spatial solitons in nematic liquid crystals: from bulk to discrete. *Optics Express* **15**, 5248–5259 (2007).URL https://www.osapublishing.org/oe/abstract.cfm?uri=oe-15-8-5248. Publisher: Optical Society of America.

17. Raza, N., Afzal, U., Butt, A. R. & Rezazadeh, H. Optical solitons in nematic liquid crystals with Kerr and parabolic law nonlinearities. *Optical and Quantum Electronics* **51**, 107 (2019). URL https://doi.org/10.1007/s11082-019-1813-0.

18. Russell, P. S. J., Hölzer, P., Chang, W., Abdolvand, A. & Travers, J. C. Hollow-core photonic crystal fibres for gas-based nonlinear optics. *Nature Photonics* **8**, 278–286 (2014). URL https://www.nature.com/articles/nphoton.2013.312.
